# Developmental Pathways Underlying Lung Development and Congenital Lung Disorders

**DOI:** 10.3390/cells10112987

**Published:** 2021-11-02

**Authors:** Inês Caldeira, Hugo Fernandes-Silva, Daniela Machado-Costa, Jorge Correia-Pinto, Rute Silva Moura

**Affiliations:** 1 Life and Health Sciences Research Institute (ICVS), School of Medicine, University of Minho, 4710-057 Braga, Portugal; ines.in.caldeira@gmail.com (I.C.); hugomiguelfsilva@gmail.com (H.F.-S.); danielamachado81198@gmail.com (D.M.-C.); jcp@med.uminho.pt (J.C.-P.); 2 ICVS/3B’s—PT Government Associate Laboratory, 4710-057 Braga/Guimarães, Portugal; 3 PhDOC PhD Program, ICVS/3B’s, School of Medicine, University of Minho, 4710-057 Braga, Portugal; 4 Department of Pediatric Surgery, Hospital of Braga, 4710-243 Braga, Portugal

**Keywords:** congenital pulmonary airway malformation (CPAM), bronchopulmonary sequestration, bronchogenic cysts, congenital diaphragmatic hernia (CDH), congenital malformations

## Abstract

Lung organogenesis is a highly coordinated process governed by a network of conserved signaling pathways that ultimately control patterning, growth, and differentiation. This rigorously regulated developmental process culminates with the formation of a fully functional organ. Conversely, failure to correctly regulate this intricate series of events results in severe abnormalities that may compromise postnatal survival or affect/disrupt lung function through early life and adulthood. Conditions like congenital pulmonary airway malformation, bronchopulmonary sequestration, bronchogenic cysts, and congenital diaphragmatic hernia display unique forms of lung abnormalities. The etiology of these disorders is not yet completely understood; however, specific developmental pathways have already been reported as deregulated. In this sense, this review focuses on the molecular mechanisms that contribute to normal/abnormal lung growth and development and their impact on postnatal survival.

## 1. Overview

Lung development is a highly orchestrated and conserved multistage process sustained by molecular, cellular, and physical events, traversing all gestational ages. Proper lung formation relies upon the crosstalk between epithelial and mesenchymal compartments, which controls the temporal and spatial distribution of a multitude of factors and diffusible signals. Key signaling pathways play a role in this process, for instance, Fibroblast Growth Factor (FGF) [1], Retinoic Acid (RA) [2], Sonic Hedgehog (SHH) [3], Wingless-related Integration Site (WNT) [4], Transforming Growth Factor β (TGFβ), Bone Morphogenetic Protein (BMP) [5], Hippo [6], to name a few.

Congenital lung malformations arise due to abnormal embryonic development caused by an impairment of signaling and/or genetic factors. The following sections will address the key regulatory factors involved throughout the different lung developmental stages and the molecular determinants of the most common lung congenital anomalies.

## 2. Lung Development

Lung development is divided into five morphological stages that establish molecular and structural transitions: (1) embryonic; (2) pseudoglandular; (3) canalicular; (4) saccular; (5) alveolar. Due to the non-synchronous development of the lung, the timing of the stages overlaps.

Primordial lung buds originate as outpunches of the primitive foregut endoderm, and the bronchial tree is generated by reinterred budding and branching of epithelial tubules [7]. The human lung epithelium derives from the endoderm, whereas the surrounding mesenchyme originates from the mesodermal germ layer [8]. Blood vessels arise from mesodermal cells surrounding the tips of branching tubules (vasculogenesis), or through migration of blood vessels from the aortic arches into the lung, by sprouting of the pulmonary artery (angiogenesis) [9]. As the lung develops, the airways and vasculature develop, fusing at the distal end of the bronchial tree to generate millions of alveolar gas exchange units. The intricate branching pattern of the airways guarantees that, during postnatal life, humidified and cleared air is uniformly distributed to alveolar units.

### 2.1. Embryonic Stage

The embryonic phase of human lung development is characterized by the formation of two primordial right and left buds that emerge from the foregut endoderm. Lung specification begins around the fourth post-conception week (pcw) in humans, at embryonic (E) day 9.5 in mice and E11 in rats, with a localized expression of NKX2.1 (also known as TTF1, thyroid transcription factor) in endodermal cells of the ventral anterior foregut [10,11].

The epithelial outgrowth of the primary buds requires complex interactions between local signals in the prespecified foregut endoderm and inductive paracrine signals from the surrounding mesoderm, both tightly regulated in dose, time, and space [12]. Defects in these molecular players impair foregut separation and cause abnormalities in epithelium and mesenchyme differentiation. WNT signaling, particularly WNT2 and WNT2B, plays an essential role in specifying NKX2.1 respiratory endoderm progenitors in the ventral anterior mesoderm surrounding the anterior foregut endoderm [13,14]. On the other hand, WNT2/2B expression is regulated by HOXB5 [15]. The ability of WNT/β-catenin to induce NKX2.1 endoderm progenitor fate depends on the activation of BMP signaling [16]. BMP4 is expressed in the mesenchyme surrounding the anterior foregut and appears to have an inhibitory action on the transcription factor SOX2 (which promotes esophageal fate), allowing NKX2.1 expression [16]. SHH expression in the endoderm regulates BMP4 expression in the mesoderm through different downstream targets, such as FOXF1, GLI1, and GLI3. In its turn, SHH is regulated by mesodermal RA signaling [17].

**Figure 1:**
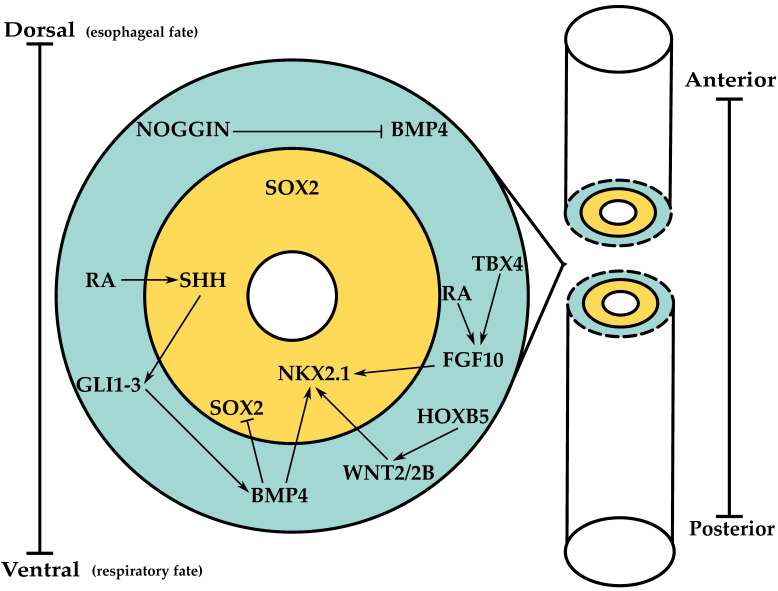
Summary of the molecular players involved in lung specification. A BMP gradient elicits dorsal SOX2 expression (esophageal progenitors) vs. ventral NKX2.1 expression (respiratory progenitors). Yellow, endoderm; blue, mesoderm.

Upon respiratory lineage specification, localized mesodermal expression of fibroblast growth factor 10 (FGF10), adjacent to the NKX2.1+ progenitors, induces FGF receptor 2b (FGFR2B) signaling resulting in primary lung bud formation due to cellular movements into the mesenchyme towards the FGF10 source. An RA-TGF-FGF10 axis triggers primary lung bud formation. Briefly, endogenous RA inhibits TGFβ signaling in the lung-specified foregut to elicit FGF10 expression and, consequently, induction of primary lung buds [18,19]. TBX4, a member of conserved T-box-containing transcription factors, influences FGF10 expression, particularly in chicken embryos. Mesodermal *tbx4* stimulates *fgf10* expression, defines its anterior-posterior (AP) mesodermal expression boundaries, and induces endoderm differentiation by triggering *nkx2.1* expression [20]. However, the genetic inactivation of TBX4 in mice does not prevent lung bud formation, suggesting a redundant role of these genes in foregut morphogenesis [21]. Figure 1 provides a schematic representation of the key genes involved in lung specification.

These early events of pulmonary development require the interplay of many other factors. For instance, the GATA family of zinc-finger transcription factors, GATA4 and GATA6, and FOXA1/2 transcription factors (Forkhead box gene superfamily) are expressed early in the endoderm and have a crucial role in survival, differentiation, and morphogenesis of the foregut [22,23,24]. Interestingly, these factors have pivotal roles in the formation of primary structures of the future lung (for instance, SOX2 and GATA6); they are also entangled in later phases of differentiation and specification of respiratory cell lineages.

Once primary lung buds are formed, epithelial tubules undergo extensive branching morphogenesis that culminates in the formation of the bronchial tree and the future alveolar region.

### 2.2. Pseudoglandular Stage

During the pseudoglandular phase (5–17 pcw human, E12–16.5 mouse, and E13–E18.5 rat), each main lung bud initiates a repetitive process of outgrowth, elongation, and bifurcation of the airway epithelium into the surrounding mesenchyme, a process called branching morphogenesis. This process is repeated over several generations; a reiterated combination of three processes, domain branching, planar bifurcation, and orthogonal bifurcation, will give rise to the respiratory bronchial tree [25,26]. Blood vessel development occurs concomitantly with epithelial branching, and vessels start to run along the airway, except that vessels branch more slowly [27]. Simultaneously, the proximal-distal axis of the developing lung is established. Additionally, as the airway tree is laid down, it begins to differentiate with cartilage; mucous glands and smooth muscle are already present [28].

Branching mechanisms are governed by intensive crosstalk between the epithelial and mesenchymal compartments, which in turn is regulated by a network of signaling cascades that control cellular processes, including extracellular matrix remodeling, proliferation, and differentiation in a temporal-spatial manner. Lung bud outgrowth, branching, and subsequent bud arrest result from the dynamic activity of SHH, FGF10, Sprouty 2 (SPRY2), TGFβ, and BMP4 [12,29].

FGF signaling, in particular FGF10, is essential for branching morphogenesis; in fact, *fgf10*^−/−^ mice die at birth due to complete abrogation of pulmonary branching morphogenesis [30]. FGF10 is expressed in the distal lung mesenchyme, specifically at sites of branch point formation and outgrowth; it acts in a paracrine fashion on the adjacent epithelium, where its cognate receptor FGFR2 is expressed [31,32], prompting a signaling cascade that culminates in the expression of SPRY2, SHH, and BMP4, which subsequently regulate the response and outgrowth of the lung bud [33,34]. This interaction is repeated each time a new series of bud initiation and outgrowth is initiated—the buds that branch from the preexisting ones grow towards the regions of high FGF10 expression [35]. Recently, there has been emerging evidence for a critical role of mammalian target of rapamycin (mTOR) complexes in coordinating branching morphogenesis and defining organ structural complexity [36]. FGF10/ FGFR2b downstream signaling through ERK1/2 (extracellular regulated kinase 1/2) and mTORC1, which is active in the epithelial progenitor cells at the tip of the airway tube, induce outward growth towards FGF10 induction signaling, controlling the duration of this event (a determinant of branch length and patterning of the airway tree) [37]. Furthermore, though the mechanisms that link vascular growth to airway branching remain unknown, it is recognized that mTORC1 directly links the primary cues for airway (FGF10) and vascular (VEGF-A; vascular endothelial growth factor A) growth in the pulmonary branching morphogenesis program [38]. The secretion of VEGF from the branching epithelium initiates the differentiation of mesenchyme progenitor cells into vascular tissue and is regulated by hypoxia-inducible transcription factors (HIFs). From the three HIF-α isoforms expressed in the developing lung, HIF-1α, present in the branching epithelium, plays a major role in early pulmonary vasculogenesis [39]. Land et al. demonstrated that FGF10 induces mTORC1 activity via SPRY2 in fetal airway epithelium, and that amplifies HIF-1α vasculogenic activity to drive VEGF-A expression and secretion from the airway endoderm [37]. The RA pathway has also been recognized as a crucial regulator for correct lung formation in mammalian [40,41] and chicken models [42]. Impairment of the RA signaling (by interfering with RDH10 or RALDH2) undermines branching morphogenesis and, thus, lung formation [43,44]. Likewise, WNT signaling is also a key regulator of lung branching morphogenesis [45,46]; targeted deletion of β-catenin [47] or the expression of the Dickkopf-1 (DKK1) WNT antagonist hamstrings normal lung branching [48]. Ligands such as WNT5A, WNT2A, and WNT7B play crucial roles in lung epithelial and mesenchymal growth/differentiation through a WNT-FGF crosstalk [49,50,51,52].

Branching morphogenesis also depends on the timely expression of numerous transcription factors. Among the subgroup of homeobox-containing genes (HOX family), HOXB5 is essential for establishing AP airway patterning. *hoxb5* is highly expressed during the pseudoglandular period, in the mesenchymal compartment, surrounding active branching sites [42,53]. As branching morphogenesis is completed and lung development progresses, *hoxb5* expression diminishes. Additionally, *sox2* and *sox9,* belonging to the SRY-related HMG-box family of transcription factors, regulate cell specification and differentiation. In the mouse, the proximal-distal patterning of the respiratory tree is defined by a distinct expression of SOX9 (and ID2) in the distal epithelium and SOX2 in the proximal airway epithelium. SOX2 is restricted to the pulmonary epithelium and is undetectable at the tips of the emerging secondary bronchi, whereas SOX9 expression delimits branching epithelium and originates distal pulmonary cell lineages [54]. In fact, it has been shown that loss of *sox2* expression at branching sites is required for branching morphogenesis to occur [55]. SOX2-SOX9 spatial distribution along the lung epithelium is regulated by FGF10 signaling; FGF10 induces *sox9* expression in the distal epithelium thus maintaining SOX9+ multipotent progenitor cells in an undifferentiated and stimulating their self-renewal; conversely, FGF10 inhibits *sox2* expression, and proximal cellular fate, in the distal lung [56]. Furthermore, loss of SOX2 signaling impairs the differentiation of secretory and multiciliated cells that line the proximal airway epithelium in the subsequent lung stage [57].

More recently, the Hippo effectors YAP (Yes-associated protein) and TAZ (transcriptional coactivator with PDZ-binding motif) have been described as having important growth-modulator functions in branching morphogenesis and epithelial cell differentiation. YAP functions at the transition zone between the airway and the distal lung compartments and controls *sox2* expression, thus promoting proximal airway differentiation [58,59]. In the absence of YAP, epithelial progenitors are unable to respond to local cues and, consequently, control SOX2 levels and spatial distribution and properly form airways [59]. Furthermore, Volckaert and colleagues proposed that cytoplasmatic YAP activity in the proximal epithelium promotes epithelial lineage commitment by inhibiting the β-catenin signaling pathway and FGF10 [60].

**Figure 2:**
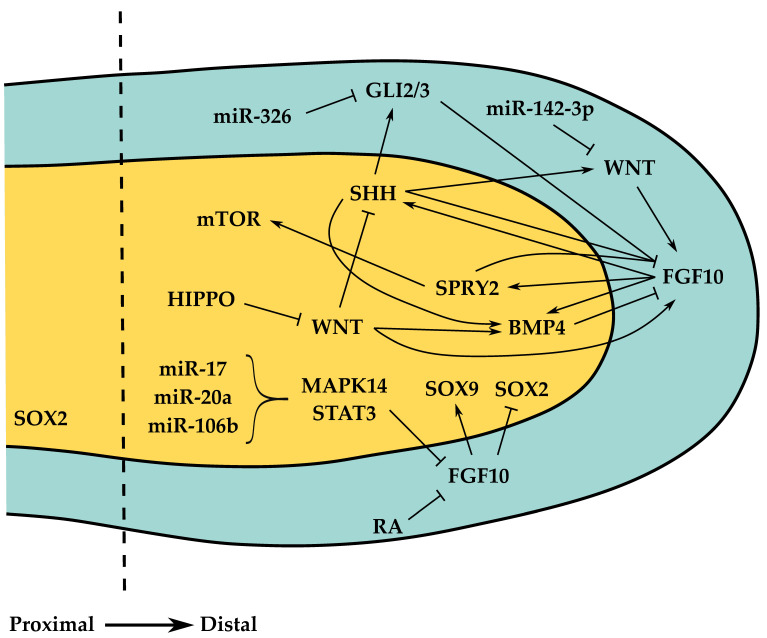
Signaling pathways that mediate epithelial–mesenchymal interactions during pseudoglandular stage, specifically in the distal epithelial tip. SOX2+ cells define proximal epithelial cell lineages whereas SOX9+ cells define distal epithelial cell lineages. Yellow, epithelium; blue, mesenchyme.

MicroRNAs (miRNAs/miR) also contribute to the lung developmental program. For instance, in this stage, miR-326 negatively regulates SHH signaling by targeting GLI2 and SMO (Smoothened) [61]. miR-142-3p regulates the proliferation and differentiation of mesenchymal progenitors by controlling the level of WNT signaling [62]. Mouse miR-17 (and its paralogs miR-20a and miR-106b) is expressed in the epithelium and controls branching morphogenesis through an FGF10-mediated molecular mechanism targeting MAPK14 and STAT3 [63]. Overexpression or deletion of the miR17–92 cluster (belonging to the miR-17 family) leads to increased proliferation/decreased differentiation or hypoplastic lungs, respectively, pointing to a crucial role in lung branching morphogenesis [64,65]. Moreover, *miR-200b^−/−^* mice display disturbed airway distal lung branching and impairment in lung parenchyma [66]. miR-449a is crucial in the pseudoglandular to canalicular transition, and its inhibition results in increased *mycn* and *sox9* mRNA and Ki-67 and SOX9 protein levels [67]. Furthermore, overexpression of rat miR-127 results in decreased terminal number of buds and increased terminal and internal bud sizes, suggesting an important role during lung morphogenesis [68]. In chicken, the expression pattern of miRNA processing machinery *drosha*, *dgcr8*, *exportin-5,* and *dicer1* was described, thus supporting the importance of these regulatory elements during the early stages of branching morphogenesis [69]. Branching morphogenesis is also highly influenced by biomechanical forces, such as the transmural pressure in the chest cavity and smooth muscle contractions that influence the synchronization of the branching events [70]. HIPPO/YAP signaling regulates myosin light chain kinase activity, creating mechanical forces that influence cell shape required for branching [71]. In addition, recent data highlighted the importance of metabolic regulation during the early stages of lung branching morphogenesis [72]. The key signaling pathways underlying branching morphogenesis are shown in Figure 2.

Furthermore, the extracellular matrix (ECM) undergoes a series of remodeling events for the branching process to occur. ECM composition/structure plays a decisive role in lung development and organ architecture; it directly affects the availability and activity of soluble factors (as, for instance, FGF) by influencing their diffusion rates and the accessibility of their receptors [73]. ECM components, particularly fibronectin, laminin, and collagen, actively participate in the epithelium and mesenchyme interactions by accumulating within the clefts that mark the branch points. Fibronectin deposition possibly regulates cellular migration by fixing some cells at the cleft, while the un-fixed ones can migrate and proliferate distally [74]. By the end of the pseudoglandular phase, the complete human airway structure has been established, and airway epithelial differentiation is proceeding.

### 2.3. Canalicular Stage

**Figure 3:**
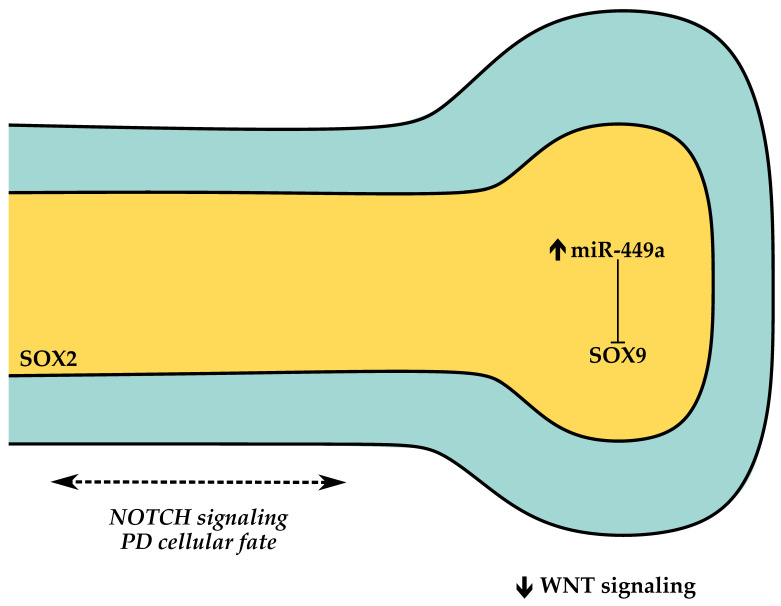
Simplified representation of the signaling pathways implicated in the canalicular stage. Proximal SOX2+ cells generate conducting airway cells (neuroendocrine, secretory, multiciliated and basal). Distal SOX9+ cells generate alveolar epithelial cells.

The canalicular stage spans pcw 16–26 in human (E16.5–E17.5 in mouse and around E18.5–E20 in rat) [25]. During this period, the existing epithelial airways continue to increase in size, and the epithelial terminal buds project into the distal airspaces as their surrounding mesenchyme thins, giving rise to the primitive pulmonary acini (terminal sacs), the primitive alveoli. This phase is also characterized by alveolar cellular differentiation. Distal epithelial cells differentiate into alveolar epithelial cells type 1 (AEC1) and type 2 (AEC2), also known as type 1 and type 2 pneumocytes. AEC2 are responsible for surfactant production and serve as AEC1 progenitor cells, whereas AEC1 are responsible for gas exchange. One of the key signaling pathways for the determination of cellular fate is NOTCH signaling. NOTCH transmembrane receptors mediate communication between neighboring cells and have a central role in balancing differentiation of multiciliated vs. secretory lineages in the proximal airway epithelium; loss of NOTCH signaling leads to the absence of secretory cells and airways populated by multiciliated cells [75]. HIPPO signaling ceases branching morphogenesis and promotes alveolar differentiation through degradation of β-catenin in the epithelium, disrupting the WNT-FGF feedback loop and directing bud tip epithelial progenitors to differentiate [76]. Finally, both human and mouse miR-449a are upregulated in the distal lung, throughout the canalicular stage, to promote distal epithelium differentiation by regulating N-MYC and SOX9 but has no effect in SOX2 expression [67]. Figure 3 illustrates the signaling events governing the canalicular stage.

Lastly, vascularization begins during this phase. The course of capillaries leaning against the distal epithelium airspaces and the continued angiogenic process contribute to the formation of the first thinned air-blood barrier in the future alveolar ducts and saccules [77].

### 2.4. Saccular Stage

The saccular phase, occurring between pcw 24 and 38 in humans (between E17.5–Postnatal day (P) 4 in mouse and E21–P4 in rat), is an intermediate phase in which branching morphogenesis ceases, and alveolarization is yet to commence. This intermediate stage is necessary since branching morphogenesis and alveolarization do not occur simultaneously [12]. Furthermore, this period is characterized by the widening and further division of the distal airspaces into numerous thin-walled terminal saccules, the alveoli precursors. The expansion of the future gas exchange region causes the condensation of the mesenchyme in-between airspaces, originating a thick immature primary septum at locations where two airspaces meet. [25]. Capillary networks remain very close to septal surfaces and are separated by a dense central layer of mesenchyme that forms a core of connective tissue [25]. During this period, the exceptional expansion of the prospective respiratory airspaces leads to a decrease in the interstitial tissue, which greatly impacts capillary arrangement. As each saccule further increases in size, blood vessels become closely associated and form a capillary bilayer wrapping each saccule. This process is crucial for alveoli formation and subsequent gas exchange [8]. Compared to the mature lung, the capillaries are embedded in a broad interstitial layer that is very poor in extracellular fibers but is very cellular [8]. Nonetheless, soon after, elastin starts to be deposited under the epithelium, which prepares the lungs for further alveolar formation.

The primary septa’s surface is mainly covered by AEC1 and AEC2, which continue to differentiate and populate the distal tubules. The surfactant system of AEC2 maturates and lamellar bodies appear, and surfactant secretion is detected [25]. With the development and maturation of the surfactant system during the saccular phase, the chances of survival to early premature infants’ increase. [78].

**Figure 4:**
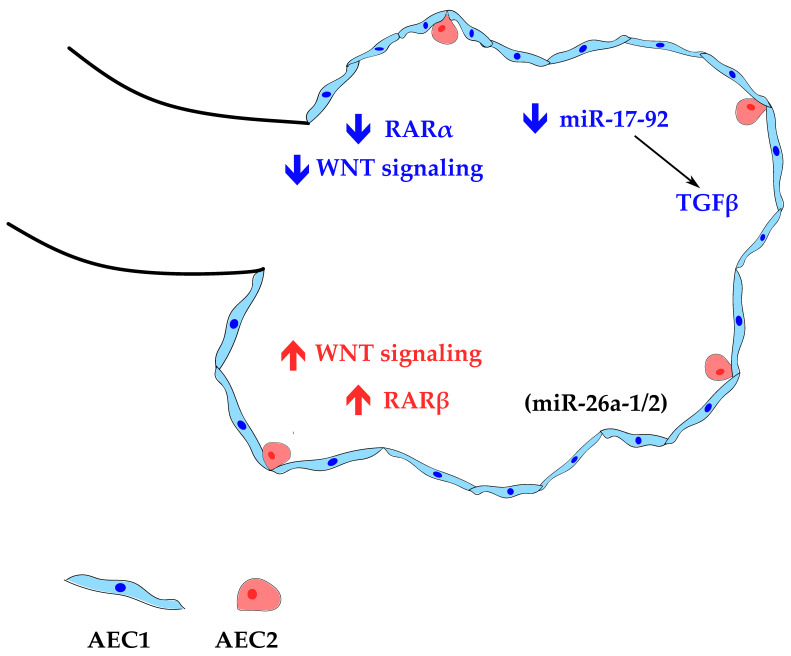
Simplified scheme of the signaling pathways involved in alveolar epithelial cell type 1 (AEC1) and type 2 (AEC2) differentiation during the saccular stage. Blue label: AEC1 differentiation; red label: AEC2 differentiation. (miR), present in the lung compartment.

RA signaling pathway seems to play an important role in preparing the lungs for sacculation and, later, in preparing for alveolar formation. Throughout the saccular stage, expression levels of the RA receptor-alpha (RARα) transcript decrease, which seems to be essential for sacculation and differentiation of mature AEC1. RA receptor-beta (RARβ) expression increases significantly in the saccular phase, matching AEC1 and AEC2 induction, pointing to a plausible role in preparing the lungs for alveolarization [79]. Similarly, loss of WNT signaling has been proven to be required to facilitate the transition from canalicular to saccular phase and allow air sac formation in the canalicular-saccular stages [80]. Interestingly, activation of WNT signaling results in the expansion of AEC2, whereas its inhibition constrains AEC2 development and shunts alveolar epithelial development toward the AEC1 cell lineage. These findings revealed that a wave of WNT-dependent AEC2 expansion is required for lung alveologenesis and maturation [81]. Moreover, studies from YAP/TAZ loss-of-function mouse mutants have shown that HIPPO signaling pathway plays a key role in promoting AEC1 fate [82]. miR-26a-1/miR-26a-2 knockout mice exhibit alterations in the morphology of distal epithelial cells, and display an increase in AEC2 cell number in the saccular stage with a concomitant increase in surfactant production; this study points to a role of miR-26a in lung maturation [83]. miR-127 expression levels are highest during the saccular/alveolar stage, and its spatial distribution shifts from mesenchymal to epithelial cells, suggesting a role in the cellular reorganization and differentiation of alveolar epithelial cells [68]. During sacculation, miR-17-92 should be repressed by HDAC3 to allow proper TGF-β signaling crucial for AEC1 remodeling [84]. Figure 4 displays the main signaling events in this phase.

### 2.5. Alveolar Stage

The final alveolar stage refers to the process of alveoli formation (alveologenesis, also known as alveolization) that gives rise to the functional units for gas exchange. The timing of alveolar development varies among species. In humans, some alveoli are already formed before birth, and this process continues postnatally until young adulthood, whereas in mice, it is mainly a postnatal process. Alveologenesis can be divided into classical (or bulk) alveolarization (week 36 of gestation—~3 years), continued alveolarization (birth-young adulthood), and microvascular maturation (week 36 of gestation—young adulthood) [25].

During classical alveolization, new secondary septa, risen from the pre-existing septa, grow from the saccular walls to subdivide the distal saccules into smaller units, the alveoli, broadening the surface area for gas exchange [28]. Like the primary septa, the secondary septa initially display a double capillary network [85]. Endothelial cells, myofibroblasts progenitors, fibroblasts, and lipofibroblasts cover the secondary septa, and matrix elastic fibers are deposited at the tip of the crests [85,86].

Classic alveolarization starts rather rapidly, but as it switches to continued alveolarization, the rate of increase in the number of alveoli declines [87,88]. Moreover, throughout postnatal lung development, the size and surface area of alveoli remain singularly stable in mice and humans. Simultaneously, microvascular maturation occurs, and the double-layered capillary network observed in the immature primary and secondary septa fuse into a more efficient single-layered one in a thin septum [25]. This process involves multi-focal fusing of capillary segments and preferential growth of the mature single-layer capillary network [85]. Since it is now widely recognized that alveolarization continues until young adulthood, the timing of microvascular maturation was re-examined. Based on stereological estimations performed during rat lung development, microvascular maturation occurs in parallel to alveolarization and continues as long as new septa or alveoli are formed [25]. Once the alveolus has matured, every AEC1 has become in close contact with the endothelium. Reciprocal crosstalk between the airway epithelium and the vascular endothelium has been investigated in mice and found to be crucial for the prosperous development of one another. In pericytes, which are mesenchymal cells that strongly interact with endothelial and epithelial cells, mechanosensitive YAP1/TAZ signaling is stimulated to release hepatocyte growth factor (HGF), which is important for effective secondary septation [89]. In addition, the respiratory epithelium is a source of alveolar epithelium-derived VEGF-A that promotes vascularization [90].

Alveologenesis involves extensive cellular and tissue remodeling that culminates in the establishment of a large gas exchange surface area. Remodeling processes include the final specification and maturation of AEC2, surfactant synthesis, AEC1 flattening, and mesenchymal differentiation. Several signaling events are associated with the processes of secondary septation and alveolarization. For instance, platelet-derived growth factor A chain (PDGF-A) and its receptor (PDGFRα/β) have been shown to play key roles in myofibroblast differentiation and production of elastin [91]. In PDGF-A deficient mice, alveolarization failed to occur and exhibited reduced elastic fibers deposition, showing that the appearance of elastin and alveolar crests are closely linked [92]. Bundles of elastin laid down by myofibroblasts restrain the differentiating alveolar cells that are expanding into sacculi as development proceeds. This remodeling process is driven by mesenchymal cells, such as the alveolar myofibroblasts (MYF), which are stimulated by PDGF-A and SHH. Furthermore, the contractibility of the MYFs physically shapes the alveolus. Additionally, WNT-responsive AEC2 receives WNT ligands from the mesenchymal cells and proliferate during this time, increasing surfactant production, which is crucial for the shift to air breathing [81].

**Figure 5:**
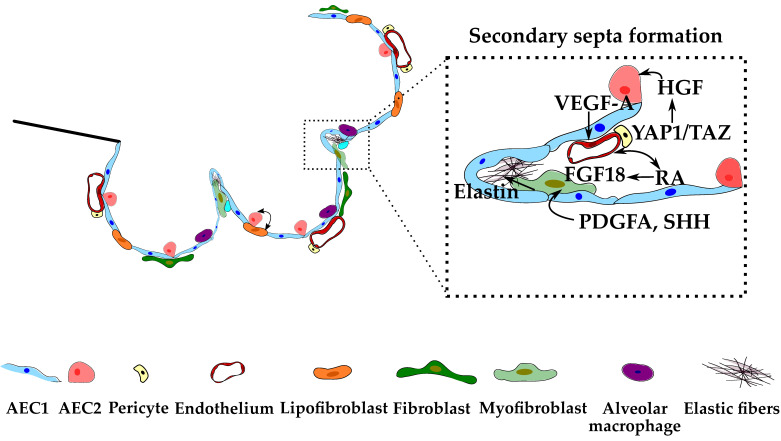
Schematic representation of the signaling events occurring during alveologenesis, particularly secondary septa formation. Left image: alveolar niche. Right image: magnification of the secondary septa.

Ephrin-B2 has been shown to play an important role in endothelial cells. Loss of ephrin-B2 signaling impairs the normal development of secondary septa and disrupts the deposition of several matrix proteins [93]. Additionally, the TGF superfamily of transforming growth factors has received great attention as a mediator of normal and aberrant lung alveolarization [94,95]. BMP, the alternative branch of the TGF-β superfamily, is also implicated in postnatal lung maturation, particularly surfactant production in neonates during respiratory adaptation to the extrauterine environment [96]. Furthermore, evidence from *fgfr3*/*fgfr4* double null mice points to a critical role for FGF signaling in controlling the alveolarization process since these animals fail to undergo secondary septation [97]. Moreover, these animals did not present the typical downregulation of elastin production that occurs at the end of this process. Finally, the retinoids have been recognized as alveolar morphogens, especially RA, which is crucial for alveolar formation [85,86]. Both increased and decreased RA signaling impair alveolar development. Several studies have demonstrated an association between vitamin A deficiency and major histological alterations, including thinner alveolar walls, airspace enlargement, and an increase in alveolar breaks [98]. On the other hand, increased epithelial RA signaling by transgenic expression of a dominant active RARα resulted in lung immaturity and a blockage in distal epithelial maturation, preventing the appearance of AEC1 cells [79]. Despite all evidence, the precise mechanism by which RA functions to regulate alveolarization is not entirely understood. Notwithstanding, the paracrine regulation of lung myofibroblast proliferation and elastin synthesis by RA is dependent on the interaction with FGF signaling, specifically FGF18 [99]. RA also acts in an autocrine manner to regulate proliferation and tube formation in endothelial cells [100]. The main interactions eliciting alveologenesis are displayed in Figure 5.

Finally, it has been demonstrated that targeting miR-34a partially improves alveologenesis in the hyperoxia-induced alveolar impairment mice model [101]. miR-29b supplementation improves alveolarization in mice that were exposed to neonatal hyperoxia and maternal inflammation [102]. miR-876-3p gain of function improved alveolar structures in the bronchopulmonary dysplasia mice model [103]. Mice miR-421 inhibition improves bronchopulmonary dysplasia condition by targeting *fgf10* [104].

## 3. Congenital Lung Malformations

Congenital lung malformations arise during development and include numerous anatomical anomalies of the lung and respiratory tree. They are usually detected prenatally by ultrasonography and comprise congenital pulmonary airway malformation (CPAM), bronchopulmonary sequestration (BPS), bronchogenic cysts (BC), and more rarely bronchial atresia, congenital lobar emphysema (CLE), and congenital tracheal obstruction. This section focuses on the molecular and genetic determinants of the most frequent anomalies: CPAM, BPS, and BC. Congenital diaphragmatic hernia (CDH) is not usually included in this group; however, since the lung is also highly affected in this condition, we have also incorporated evidence related to lung hypoplasia.

### 3.1. Congenital Pulmonary Airway Malformation

Congenital Pulmonary Airway Malformation (CPAM), previously known as Congenital Cystic Adenomatoid Malformation (CCAM), is a rare but clinically significant developmental disorder. CPAMs are the most common congenital lung abnormalities, with an estimated incidence between 1 in 25,000–35,000 live births [105]. However, recent data and reports from the European Surveillance of Congenital Anomalies (EUROCAT) suggest a much higher prevalence of this disorder [106,107]. CPAMs are associated with significant infant morbidity and mortality due to associated complications such as lung hypoplasia, respiratory distress, and fetal hydrops [108,109].

CPAM is characterized by dilation of the airways and consequent cystic lesions within the lung parenchyma; it displays a disorganized spatial arrangement of tissues where multicystic masses replace the normal lung and are connected to the tracheal-bronchial system. Most of the cases involve a single pulmonary lobe, and bilateral lesions are uncommon. Stocker’s classification subdivide CPAM into five types based on clinical, macroscopic, and microscopic criteria [110,111]. CPAM type 1 is the more frequent cystic lesion (~60% frequency) and comprises multiple large cysts (2–10 cm) or a single dominant cyst. In this case, cysts are lined by ciliated pseudostratified columnar epithelium, and the walls are composed of fibromuscular connective tissue with cartilage presence, in some cases. CPAM type 2 lesion (~20% frequency) consists of smaller cysts (0.5–2 cm) with a sponge-like appearance. Cystic structures are lined by ciliated cuboidal or columnar epithelium, and walls are formed by a small portion of fibromuscular connective tissue. CPAM type 3 (~10% frequency) is characterized by multiple microscopic cysts (0.5 cm) with an adenomatoid appearance, resembling a bronchiolar structure. The more recently added subtypes are CPAM type 0 and type 4. CPAM type 0 (~2% frequency), also known as acinar dysplasia, consists of solid lesions with tracheal or bronchial-like structures composed of cartilage and smooth muscle. Lastly, CPAM type 4 (~10% frequency) or distal acinar-alveolar malformation consists of varying-sized cysts lined by type 1 and type 2 alveolar cells. All five CPAM subtypes originate from different locations of the pulmonary airway structure, from proximal tracheobronchial (type 0) to bronchial/bronchiolar/alveolar (type 1, 2, 3), to distal acinar (type 4) [109,110,112].

To perform an antenatal practical evaluation of CPAMs, Adzick’s classification system based on gross anatomy and sonographic appearance is widely accepted. This classification has a relevant degree of prognosis and distinguishes between microcystic and macrocystic lesions [113]. Macrocystic lesions are composed of a single or several large cysts (≥0.5 cm) and appear as fluid-filled structures in the ultrasound. On the other hand, microcystic lesions are smaller (≤0.5 cm), solid, and bulky. While microcystic lesions are associated with a poor prognosis due to associations with hydrops, macrocystic lesions usually have a more favorable prognosis [113]. Nonetheless, several other classification systems have been proposed throughout the years [114].

The pathogenesis of CPAM remains uncertain, although it is believed that defective airway proximo-distal patterning and abnormal lung branching are associated with pulmonary cysts formation [108,114]. Some authors consider CPAM a hamartomatous abnormality, while others hypothesize that CPAM is caused by a focal arrest of lung development during different stages of branching morphogenesis. Numerous molecular mechanisms have been explored as potential contributors to CPAM etiology [112,114,115]. For instance, altered cellular processes such as increased cell proliferation and decreased apoptosis are typically associated with CPAM lesions [116]. The protein levels of cell adhesion molecules such as Integrin and E-cadherin are altered in CPAMs, suggesting uncharacteristic cytoplasmic signaling [117]. Platelet-derived growth factor (PDGF-BB) has maximal activity in the canalicular phase, stimulating lung growth by increasing proliferation but later, in the saccular stage, PDGF-BB action decreases. In utero resected CPAM lesions with rapid growth and associated hydrops show high PDGF-BB mRNA and protein expression levels. Moreover, PDGF-BB has a high and specific expression in the mesenchymal compartment of epithelial-lined cysts [118]. Glial Cell-Derived Neurotrophic Factor (GDNF) is expressed in the epithelial and endothelial compartments during lung organogenesis but is absent postnatally. Conversely, in postnatal resected CPAMs, GDNF is highly expressed in the epithelium suggesting a dysregulation of the GDNF signaling pathway [119]. Decreased mRNA and protein levels of Fatty Acid-binding Protein-7 (FABP-7) are observed in CPAM specimens compared to normal fetal lungs, suggesting a potential decrease in glucocorticoid response in CPAM lesions [120]. In addition, Clara Cell marker 10 (CC10) overexpression is observed in CPAM cysts [115]. Also, KRAS signaling and PI3K-AKT-mTOR pathway may play a role in the pathogenesis of CPAM lesions [121]. Increased protein expression of Vascular Endothelial Growth Factor Receptor 2 (VEGFR2) is observed in postnatal CPAM, compared to normal lung, pointing to a role for the VEGF system in these congenital lesions [122].

An imbalance of early developmental markers is also observed in CPAMs. HOXB5 transcription factor is highly expressed during the pseudoglandular period. Nonetheless, the protein expression levels decrease in the canalicular phase and, in the alveolar phase, expression is negligible [42,53]. In CPAM lesions, HOXB5 protein expression levels are increased and present in the mesenchyme adjacent to abnormal branched airways, resembling a phenotype of earlier developmental stages [123]. Likewise, TTF1 is also crucial in regulating early lung development. In CPAM lesions, TTF1 presents differential expression patterns. In CPAM types 1 and 2 TTF1 expression pattern resembles typical pseudoglandular stage lungs. On the other hand, CPAM type 4 lesion presents TTF1 spatial distribution comparable to the canalicular stage [124]. Another study detected increased *hoxb5*, *ttf1*, and *fgf9* and decreased *fgf7* expression levels in fetal CPAM lesions, but no differences were noticed for *fgf10* and *fgfr2* in both fetal and postnatal CPAM lesions [125].

FGF10 is a mesenchymal growth factor critical for the epithelial-mesenchymal interactions that occur during lung branching. Rat fetal lung localized overexpression of *fgf10* at distinct developmental stages induces CPAM-like lesions. Depending on the localization/stage, *fgf10* overexpression induces macrocystic and microcystic malformations highly similar to those observed in humans [126]. Heterotopic overexpression of *fgf7* and *fgf10* and orthotopic expression of *fgf9* in transgenic mice disrupted pulmonary morphogenesis, pointing towards a role in cysts formation [127,128,129]. Moreover, mesenchyme-free epithelial explant cultures with FGF7 supplementation promoted epithelial proliferation, leading to the formation of cyst-like structures [130]. In addition, mouse microRNA-processing enzyme DICER mutant lungs result in upregulation of mesenchymal *fgf10* expression and, consequently, lung branching arrest and large epithelial pouches (cystic-like) [131]. This mechanism may involve intermediary *shh* downregulation [132,133]. Yin Yang 1 (YY1) transcription factor lung epithelial mutations cause abrogated mouse lung branching and airway dilation comparable to human CPAMs. This phenotype can be justified by reduced *shh* expression and subsequent upregulation of mesenchymal *fgf10* in an YY1-SHH-FGF10 molecular axis [132,133]. Epithelial cell-specific deletion of small GTPase *cdc42* in fetal mice disrupts epithelial cell polarity, proliferation, and mitotic spindle orientation, resulting in dilated respiratory tubules. This phenotype was accompanied by broader *fgf10* mesenchymal expression and decreased *shh* and *ptc1* (Cell Surface Transmembrane PATCHED 1) [134]. Combined deletion of *foxa1* and *foxa2* transcription factors disrupts pulmonary branching after E12.5 and result in the formation of large cysts at E15.5 and afterward. This morphological phenotype was associated with decreased *shh* expression [24]. Loss of WNT signaling receptor Frizzled 2 (*fzd2*) in the mouse lung epithelium causes large cysts formation in the distal region of the lung. Moreover, cysts formation was associated with decreased epithelial RhoA (Transforming Protein RhoA) signaling and no impact on WNT/β-Catenin signaling. Though, increased *fgf10* expression and decreased *fgfr2* and *shh* were observed [135]. Combined mutations of Histone Deacetylases *hdac1* and *hdac2* in the developing lung epithelium resulted in defects in branching morphogenesis and cysts formation in mouse E12.5 lungs [136]. E18.5 mouse lungs with epithelial *bmpr1a* (Bone Morphogenetic Protein Receptor Type 1A) deletion develop dramatic defects, with lungs containing large-fluid spaces [137]. Similar phenotypes occur under mouse conditional deletion of the proto-oncogene *mycn* [138]. Conditional mutation of the HIPPO pathway effector *yap* also results in dilated cyst-like structures [59]. *sox2* gene has a critical role during lung branching morphogenesis. *sox2* is expressed in non-branching regions and absent from branching sites. Overexpression of SOX2 in mouse lung epithelium disrupts branching morphogenesis and results in cystic-like structures. This data suggests that forced proximal epithelial differentiation leads to the CPAM phenotype [55]. Modulating the timing of ectopic *sox2* expression of branching regions results in cystic lesions that resemble the spectrum of human CPAMs [139]. Embryonic airway epithelial markers SOX2 and TTF1 are also present in adult human CPAMs, resembling the epithelial expression of the developing lung. Additionally, the Retinoic Acid signaling component Retinal Dehydrogenase 1 (RALDH1) shows weak expression in adult CPAM lesions [140]. In mouse epithelial-specific studies, both gain and loss of function of *sox9* gene resulted in cystic-like structures at distal epithelial branch tips [54]. Transgenic Notch signaling misexpression in lung mice prevents the differentiation of alveolar cell types and results in distal abnormal cysts, which express typical proximal markers. Such data suggests defective proximal-distal patterning [141]. While many studies focus on the epithelial compartment, recent data revealed that CPAM lesions also impact the adjacent mesenchymal tissues with alterations in airway smooth muscle cells and extracellular protein products such as elastin [142]. Although significant advances have been made in understanding the molecular basis of CPAM, the pathogenesis of this congenital defect remains quite unknown. Still, CPAM is considered a unique model to study the molecular pathogenesis of isolated structural birth defects [121].

Considerable progress has been made in the diagnosis and treatment of CPAM lesions. However, the management of CPAM is still a matter of debate. Prenatal diagnosis of CPAM is crucial for the supervision of patients in the prenatal and postnatal periods. Most abnormal lung lesions are detected as early as the 20^th^ week of gestation. Fetal ultrasonography is usually used to detect lesions’ growth and potential complications and allows the calculation of the CPAM volume ratio (CVR), a value used to predict the prenatal course. When ultrasound is unreliable or inconclusive, evaluation is performed by Magnetic Resonance Imaging (MRI). Frequently, CPAMs tend to increase in size until the 28^th^ gestational week, reach a plateau, and then start to regress. Fetal intervention is necessary when there is a persistent mediastinal shift and/or hydrops develops. CPAM prenatal interventions include systemic corticosteroid therapy, thoracoamniotic shunts or single-needle thoracentesis, and fetal lobectomy by minimally invasive procedures or open surgery [108,114,143,144]. Most babies require respiratory support at birth, and postnatal examinations combine several imaging methods such as Computed Tomography (CT) and X-ray. The exact optimal time for surgery is still inconclusive, but early interventions are recommended in symptomatic offspring. Neonatal resection is recommended for symptomatic patients, while asymptomatic resection remains controversial. There are three general indications to operate in asymptomatic cases: risk of malignancy, risk of complications (such as the risk of infection and pneumothorax), and potential for compensatory lung growth with earlier resection. The optimal operative management methods use minimally invasive approaches, with thoracoscopic techniques adopted over traditional thoracotomy [108,143,144,145]. According to the existing data, no apparent decrease in lung function is observed after surgery in the short term. As for the long term, children who undergo surgery display normal exercise tolerance and similar quality of life compared to otherwise healthy children [108].

### 3.2. Bronchopulmonary Sequestration

Bronchopulmonary sequestration (BPS) is a rare congenital malformation of the lower respiratory tract characterized by a non-functioning mass of lung tissue with an anomalous arterial supply from systemic circulation (usually the aorta) not involved in lung oxygenation [146]. In this disorder, embryonic pulmonary tissue detaches from the tracheobronchial tree and then degenerates into a cyst alongside the normal developing lung. Concurrently, an abnormal connection between this cystic structure and systemic circulation occurs [147]. BPS is classified as intralobar (ILS) or extralobar (ELS), depending on their location in the lung [148]. ILS is located inside a normal pulmonary lobe and shares a common pleura. In opposition, ELS is separated by the visceral pleura and forms a separate lobe [149]. Moreover, ELS is often linked with other congenital malformations, including congenital diaphragmatic hernia (CDH), congenital bronchopulmonary foregut malformations, CPAM, congenital heart disease, pulmonary hypoplasia, vertebral anomalies, and colonic duplication [148].

Bronchopulmonary sequestration accounts for 1.1% to 1.8% of all congenital bronchopulmonary anomalies. Intralobar cysts are more common, predominantly on the left lower lobe, and are frequently diagnosed in adolescent or adult patients; failure of earlier diagnosis can lead to repeated pneumonia and hemoptysis [150,151,152,153]. Conversely, ELS is a disease confined to neonates because of the high frequency of concomitant congenital abnormalities [154,155]. Pulmonary sequestration is not believed to be familial; however, some rare cases point to the possibility of a genetic predisposition for this condition [156,157].

Human studies have established that both types of BPS are commonly associated with CPAM [158,159,160,161]. The aberrant features of BPS and CPAM lesions are thought to be due to alterations in cell adhesion mechanisms that lead to atypical cell migration and proliferation during early lung branching morphogenesis. In fact, altered α2β1-integrin signaling triggers alterations in cell-cell adhesion and, consequently, changes in epithelial cell migration and cell proliferation [117]. In addition, β1-integrin signaling is essential for the migration of epithelial cells during lung development [162]. It has been demonstrated that abnormal Hoxb5 regulation triggers alterations in branching, such as the formation and persistence of immature and dysfunctional tissue, and is associated with BPS [123,163]. Additionally, in BPS, there is a decrease in α2-integrin protein levels, which is consistent with HOXB5 downregulation and its well-known role as a regulator of integrins and E-cadherins [164].

In the past, BPS malformations were thought to be rare and were mainly seen in autopsy cases, associated with other developmental abnormalities such as CDH, hydrops, and polyhydramnios. Nowadays, BPS is identified on prenatal ultrasound, and fetus outcome depends on the location of the lesion. Lesions located below the diaphragm usually have a more favorable prognosis, while intrathoracic lesions are commonly correlated with poor prognostic due to associations to hydrothorax, which can lead to fetal hydrops, polyhydramnios, and fetal death. In this case, a thoracoamniotic shunting may be required [165]. Postnatal complications, such as respiratory distress, infection, intrathoracic bleeding, hemoptysis, cardiac failure, and the potential risk of malignancy require early surgical excision.

### 3.3. Bronchogenic Cyst

Bronchogenic cyst (BC) is a developmental anomaly that results from abnormal budding of the primitive foregut. BCs are isolated choristomas characterized by closed respiratory epithelium-lined sacs with walls composed of cartilage. Other bronchial structures can also be found in BCs, such as smooth muscle and mucous glands. BC lesions are typically unilocular and fluid or mucus-filled. Though they can locate anywhere along the foregut, they are frequently localized in the mediastinum, adjacent to the carina region. BCs can also arise in the lung parenchyma, but no communication occurs with the airways [109,143,166].

The molecular mechanisms underlying BCs are still unknown. However, it is believed that abnormal growth of the upper gastrointestinal and respiratory tracts contributes to this condition. Moreover, BCs are not typically associated with genetic or chromosomal differences [167].

Bronchogenic cysts rarely require prenatal intervention. However, occasionally, they become large and cause complications, such as external compression and resulting hydrops. Prenatal interventions to remove BC lesions include thoracentesis or thoracoamniotic shunts to relieve fluid accumulation; resection is only necessary for rare complications. Nonetheless, removing BCs in the postnatal period is preferred considering the high risk of infection associated with prenatal procedures. Still, BCs always need to be removed due to the risk of becoming malignant [109,143,166].

When asymptomatic and not detected by prenatal ultrasound, BC lesions are only detected postnatally due to an infection or mass-related symptoms (congenital lobar overinflation, dysphagia, hemothorax, respiratory distress, dyspnea, recurrent pneumonia, or hemorrhage). Correct diagnosis using X-ray, CT, and MRI and subsequent management are crucial since BCs can become malignant. Postnatal treatment consists of surgical enucleation or resection by lobectomy using Video-Assisted Thoracoscopic Surgery (VATS) whenever possible. Definite diagnosis is determined by histopathology, and recurrence is improbable after complete resection [109,143,166,168]. Children with bronchogenic cysts have normal lung function after lesions removal [167].

### 3.4. Congenital Diaphragmatic Hernia

Congenital Diaphragmatic Hernia (CDH) is a congenital condition, with a prevalence rate between 1 in 2500–3000 live births [169]. CDH is characterized by a defect in the diaphragm that leads to the protrusion of the abdominal content into the thoracic cavity, impacting lung growth and development [170]. Human diaphragm development starts approximately at the fourth week of gestation, and by week 12, it is already fully formed. Closure of the diaphragm occurs typically around the eighth week of gestation, and sealing of the left side occurs one week later than the right side [171,172]. Based on the anatomical position of the defect, CDH can be classified into the posterolateral (Bochdalek hernia), anterior (Morgagni hernia), and central hernia [172,173]. In CDH, abnormal pulmonary development is characterized by decreased terminal branching leading to hypoplasia, reduced gas exchange area, thickened alveolar walls, and increased interstitial tissue. Concurrently, the pulmonary circulation is also profoundly affected, triggering persistent pulmonary hypertension, contributing to higher mortality and morbidity [174]. Consequently, babies with CDH often suffer from cardiorespiratory failure at birth [175].

The pathogenesis of CDH is still not fully understood. Chromosomal anomalies such as aneuploidies, structural rearrangements, copy number variants, single-gene mutations, and monogenic syndromes contribute to the heterogenic etiology of CDH [176,177]. Nonetheless, only 30% of the CDH cases have been associated with genetic factors and, for this reason, several animal models have been used to study this condition. Teratogenic and genetic rodent models have contributed to disentangling CDH pathophysiology and unravelling the molecular mechanisms underlying lung underdevelopment and diaphragmatic defect. On the other hand, large experimental animal models (sheep and rabbit) have been valuable to improve surgical approaches and prenatal therapies [174,178,179].

One of the most used teratogenic models is the mouse/rat nitrofen model. Nitrofen is an herbicide (considered a 2B class carcinogen) that disrupts critical pathways for diaphragm development, lung branching morphogenesis, and alveolar differentiation, mimicking human CDH defects [178,179]. Data obtained from the nitrofen model revealed that pulmonary hypoplasia could be settled without the diaphragmatic defect. These findings lead to the proposal of the *dual-hit hypothesis*, which explains lung hypoplasia in CDH as the result of two independent developmental insults: an early insult that occurs before diaphragmatic closure and a late insult that follows the appearance of the diaphragmatic defect and, consequently, herniation of abdominal organs into the thoracic cavity [180,181].

The molecular characterization of the nitrofen model revealed that nitrofen targets several steps/components of RA signaling. For instance, it inhibits retinal dehydrogenase 2 activity (RALDH2), a key enzyme in the RA synthetic pathway, and downregulates the retinol storage enzyme, lecithin:retinol acyltransferase (LRAT), and the RA-degrading enzyme CYP26. At the genomic level, it causes mutations in the STRA6 membrane receptor for serum retinol and deletions on the 15q chromosome, which contains the encoding gene for a cellular retinoic acid-binding protein (CRABP1). Conversely, antenatal administration of RA to the nitrofen model clearly reduced CDH incidence [182,183]. Furthermore, human studies detected low retinol levels in infants diagnosed with CDH compared to non-CDH babies [184,185]. In summary, it seems that a disruption in the RA pathway could be responsible for the morphological changes seen in CDH [174,179,186,187,188].

As stated in Section 2 of this review, lung development requires a synchronized pool of several growth factors/signaling pathways to give rise to a fully functional organ. Alongside RA, other signaling pathways underlying early and late pulmonary epithelial differentiation and mesenchymal development, such as FGF, BMP, WNT, are downregulated in the nitrofen-induced CDH model, thus contributing to lung hypoplasia [186]. In some cases, these findings have been corroborated with human data from amniotic fluid [189] or lung tissue [190,191]. Additionally, transcription factors and ECM components also contribute to the etiology of diaphragmatic defects and associated lung abnormalities. For instance, GATA4, a retinoic acid-inducible transcription factor, is essential for diaphragm and lung organogenesis. Hence, the lack of GATA4 during mouse development causes CDH [192,193]. Concurrently, knockout mice for NR2F2 (formerly called COUP-TFII) and ZFPM2 (also known as FOG2), both modulators of RA transcriptional activity, exhibit diaphragmatic hernia and lung hypoplasia [194,195]. Ablation of several other RA-dependent transcription factors such as WT1, SOX7, GATA6, and MYRF leads to CDH development, causing different types of herniation and pulmonary underdevelopment [196,197,198,199]. GLI, KIF7, and PBX1 knockout mice have revealed a role for SHH signaling in CDH [200,201,202]. Knockout mice for SLIT3, ROBO1, GPC3, NDST1, FREM1, FRAS1, and FREM2 uncovered their function in different aspects of normal diaphragm development [174,179,203].

More recently, epigenetic alterations, particularly microRNAs, have been associated with CDH pathophysiology. Several miRNAs have been associated with different lung developmental stages [204]. In the CDH context, miR-200b is upregulated in both human and animal samples. Moreover, tracheal fluid from CDH survivor babies that underwent FETO (fetoscopic endoluminal tracheal occlusion) exhibited elevated levels of miR-200b when compared to non-survivors [205,206]. Furthermore, the same group demonstrated that miR-200b prenatal treatment reduces the incidence of CDH defects in the nitrofen model [207]. The authors suggest that this upsurge in miR-200b levels may result from compensatory mechanisms to promote lung maturation [208].

Despite the relatively low prevalence rate of this congenital condition, the mortality rate is considerably high. Long-term outcomes depend on the characteristics of the diaphragmatic defect [170,209], and CDH survivors have a high risk of long-term morbidities and a lower quality of life. CDH lesions can be identified by ultrasonography around the 22–24th week of gestation; prenatal MRI can also be performed to predict prenatal course [171]. Fetal interventions, such as FETO, have been associated with increased survival among some CDH-infants; however, studies are still ongoing to further determine patient risk/benefit [209,210]. Postnatal surgical intervention to repair the diaphragmatic effect is required, and the surgical approach depends on the size/type of defect. However, babies born with CDH exhibit life-threatening pulmonary hypertension that needs to be resolved before surgery. Lung-protective ventilator strategies or “gentle ventilation”, extracorporeal membrane oxygenation (ECMO), and cardiopulmonary pre-operative stabilization are strategies currently used to tackle hypertension and improve patient outcomes [211,212,213].

## 4. Concluding Remarks

During embryonic development, coordinated growth and differentiation are essential to the formation of fully functional organs. In the particular case of the lung, a continuous interplay between lung compartments is precisely regulated by distinct developmental pathways. Table 1 summarizes the key molecular signals involved in each stage of development.

A deeper understanding of the mechanisms controlling lung morphogenesis is crucial for managing congenital/neonatal respiratory diseases. Furthermore, it is highly relevant for developing new strategies to improve the regenerative response of the lung to injury. With this review, we aimed to describe the key signaling pathways underlying normal lung development. Additionally, we reviewed the current knowledge regarding the signaling events impaired in congenital lung lesions. Congenital lung lesions present several alterations in both the epithelial and mesenchymal compartments that, altogether, contribute to the abnormal lung phenotype. Table 2 summarizes the molecular players known to be altered in CPAM and CDH. Nonetheless, more studies are in demand to determine the etiology of these congenital conditions.

## Figures and Tables

**Table 1:** Summary of the molecular players and corresponding major events underlying normal lung development.

Stage	Major Events	Molecular Players	Expression Site	Function
Embryonic	Lung bud develops from the foregut; Tracheoesophageal septation	NKX2.1 (or TTF1)	Endoderm	Specification of respiratory progenitors Epithelial marker
		TBX4	Mesoderm	Induction of endodermal differentiation (*nkx2.1*-dependnent) & budding (*fgf10*-dependent)
		WNT2/2B	Mesoderm	Specification of NKX2.1 respiratory progenitors
		GATA4/6	Endoderm	Formation of primary lung structures Differentiation of visceral endoderm
		SHH/ GLI1-3	Endoderm/ Mesoderm	Embryonic foregut development
		FOXF1	Mesoderm	Lung & gastrointestinal morphogenesis Regulation of mesenchymal-epithelial interactions
		FOXA1/A2	Endoderm	Specification of foregut endoderm Branching morphogenesis
Pseudoglandular	Formation of bronchial buds: Branching morphogenesis. Formation of bronchial tree and blood vessels; Cellular differentiation (cartilage, smooth muscle);	FGF10/ FGFR2	Mesenchyme/ Epithelium	Lung bud outgrowth
		SPRY2	Distal epithelium	Negatively regulates FGF10 signaling Inhibits lung budding
		TGFβ	Mesenchyme/ Epithelium	Lung branching
		BMP4	Epithelium of distal tips	Negatively regulates FGF10 signaling Inhibits lung budding
		SHH	Epithelium	Branching morphogenesis
		RDH10	Lung buds	Lung bud outgrowth
		RALDH2	Mesothelial region	Lung growth and branching
		WNT7B	Distal epithelium	Branching morphogenesis
		WNT2A	Distal lung mesothelium	Mesenchymal cell proliferation
		WNT5A	Mesenchyme/ Epithelium	Tracheal development/ Distal lung morphogenesis
		β-Catenin	Airway epithelium	Branching morphogenesis Epithelial differentiation and proliferation
		HOXB5	Mesenchyme	Anterior-posterior patterning Directing epithelial morphogenesis
		SOX2	Proximal epithelium	Proximal-distal patterning
		SOX9	Distal epithelium	Proximal-distal patterning
		miR-17 miR-20a miR-106b	Epithelium	FGF10-mediated epithelial branching morphogenesis
		miR-200b	Epithelium & Mesenchyme	Distal airway branching
		miR-449a	Distal epithelium	Regulation of differentiation & proliferation
		miR-326	Mesenchyme	SHH signaling modulator
		miR-142-3p	Mesenchyme	Proliferation & differentiation of mesenchymal progenitors Control of WNT signaling
Canalicular	Formation of distal most airways; Alveolar cellular differentiation; Appearance of the first air-blood barrier; Surfactant production initiation	NOTCH		Determination of cellular fate Development of microvasculature network
		miR-449a	Distal epithelium	Regulation of differentiation & proliferation
Saccular	Formation of thin-walled terminal saccules (alveoli precursors); Maturation of vasculature and surfactant system	RARα	Epithelium	Sacculation & differentiation of mature AEC1
		RARβ	Epithelium	AEC1 & AEC2 induction
		miR-26a-1/ miR-26a-2	Alveolar epithelial cells	Pulmonary surfactant synthesis
		miR-17-92	Epithelium	AEC1 remodeling
Alveolar	Alveologenesis: establishment of secondary septa and alveoli formation; Microvascular maturation: single-layered capillary network formation.	VEGF-A	Epithelium	Vascular development
		PDGF-A/ PDGFRα/β	Epithelium/ Mesenchyme	Myofibroblast differentiation Elastin production Secondary septation & alveolarization
		Ephrin-B2	Microvasculature	Secondary septation & alveolarization
		miR-34a		Impairs alveolarization
		miR-29b		Promotes alveolarization
		miR-876-3p		Promotes alveolarization
		miR-421	Alveolar epithelial cells	Disrupts alveolarization

**Table 2:** Summary of the molecular players impaired in congenital lung diseases.

Congenital Lung Disease	Altered Epithelial Signaling/Expression	Altered Mesenchymal Signaling/Expression	References
**CPAM**	Integrin	Integrin	[117]
	E-cadherin	-	[117]
	-	PDGF-BB	[118]
	GDNF	-	[119]
	-	FABP-7	[120]
	CC10	-	[115]
	KRAS; PI3K-AKT-mTOR	-	[121]
	VEGFR2	-	[122]
	-	HOXB5	[123,125]
	TTF1/Nkx2.1	-	[124,125,140]
	*fgf9*	-	[125,129]
	-	*fgf7*	[125,127,130]
	-	*fgf10*	[126,128,131,132,134,135]
	*fgfr2*	-	[135]
	DICER	-	[131]
	*yy1*	-	[132,133]
	*shh*	-	[24,132,134]
	*cdc42*	-	[134]
	-	*ptc1*	[134]
	*foxa1*; *foxa2*	-	[24]
	*fzd2*	-	[135]
	RhoA	-	[135]
	*hdac1*; *hdac2*	-	[136]
	*bmpr1a*	-	[137]
	*Mycn*	-	[138]
	*Yap*	-	[59]
	SOX2	-	[55,139,140]
	SOX9	-	[54]
	Notch	-	[141]
	-	Elastin	[142]
**CDH**	-	RALDH2	[187]
	RAR	RAR	[186]
	-	STRA6	[174,187]
	Midkine	Midkine	[186]
	FGF signaling	-	-
	-	FGF10	[189]
	FGF2	FGF2	[186]
	-	FGF7	[189,190]
	FGF9	-	[186]
	-	FGF18	[191]
	FGFR2	FGFR2	[186]
	FGFR3	FGFR3	[186]
	BMP signaling	-	-
	BMP4	-	[186]
	BMP7	-	[186]
	BMPR2	-	[186]
	WNT signaling	-	-
	-	WNT2	[186]
	WNT5A	WNT5A	[186]
	WNT7B	-	[186]
	-	GATA6	[198]
	-	GATA4	[192,193]
	-	NR2F2	[194]
	ZFPM2	-	[195]
	-	WT1	[196]
	-	MYRF	[199]
	-	GLI	[200]
	-	KIF7	[201]
	-	PBX1	[202]
	-	SLIT3	[174,179,203]
	ROBO1	ROBO1	[174,179]
	NDST1	-	[174,179]
	FREM1	-	[174,179]
	-	FRAS1	[174,179]
	-	FREM2	[174]
	miR-200b	miR-200b	[207]

## Data Availability

Not applicable.
